# Origin, Differentiation, and Function of Intestinal Macrophages

**DOI:** 10.3389/fimmu.2018.02733

**Published:** 2018-11-27

**Authors:** Calum C. Bain, Anika Schridde

**Affiliations:** Centre for Inflammation Research, University of Edinburgh, Edinburgh, United Kingdom

**Keywords:** macrophage, monocyte, intestine, inflammation, colitis, ontogeny

## Abstract

Macrophages are increasingly recognized as essential players in the maintenance of intestinal homeostasis and as key sentinels of the intestinal immune system. However, somewhat paradoxically, they are also implicated in chronic pathologies of the gastrointestinal tract, such as inflammatory bowel disease (IBD) and are therefore considered potential targets for novel therapies. In this review, we will discuss recent advances in our understanding of intestinal macrophage heterogeneity, their ontogeny and the potential factors that regulate their origin. We will describe how the local environment of the intestine imprints the phenotypic and functional identity of the macrophage compartment, and how this changes during intestinal inflammation and infection. Finally, we highlight key outstanding questions that should be the focus of future research.

## Introduction

The gastrointestinal tract faces an unrivaled exposure to foreign antigens and, as a result, is home to the largest compartment of the immune system. This includes a network of mononuclear phagocytes (MPs), including macrophages and conventional dendritic cells (cDCs), that play distinct yet complementary roles in discriminating between innocuous antigens and potential pathogens, ensuring that the appropriate response is mounted to each. While this is a highly efficient process, it can break down in some individuals, leading to the development of chronic inflammation, such as inflammatory bowel disease (IBD) in which inappropriate immune responses are mounted against the commensal microbiota. Thus, there is great interest in understanding the biology of intestinal MPs. The role of macrophages in health and disease has attracted particular attention, as their plasticity and wound healing capabilities make them attractive targets for potential novel therapies to treat IBD. In this article, we will first discuss the current understanding of macrophage heterogeneity in the gut wall, before describing the roles macrophages play in intestinal homeostasis and how this may depend on their anatomical positioning. We will then review the recent developments in intestinal macrophage ontogeny, discussing how the local environment of the gut imprints the phenotypic and functional identity of macrophages, before finally describing the changes that occur when homeostasis is perturbed by inflammation.

## Identifying macrophages in the gut wall

One of the major issues that has stifled our progress on understanding the immunobiology of intestinal macrophages is their inaccurate identification. For instance, although murine macrophages have traditionally been identified based on their expression of the pan-macrophage marker F4/80 (1), it is clear that other cells, such as conventional dendritic cells (cDCs) and eosinophils can express F4/80 to some extent (2, 3). Furthermore, many macrophages, including those in the intestine, express high levels of CD11c and MHCII, markers that have classically been used to identify cDCs (3). Thus, the identification of intestinal macrophages requires a multi-parameter approach. The Mer tyrosine kinase (MerTK) and the high affinity FcγR1 (CD64), have emerged as superior markers for the identification of macrophages across different tissues (4–6), the latter also being useful across species (7–9, 10). When used in combination with CD11c and MHCII, CD64 expression distinguishes macrophages from *bona fide* cDC in the gut wall (5, 10, 11). This is corroborated by the distinct growth factor dependency and migration patterns of CD64-defined MPs. Whereas CD64^+^ MPs are highly dependent on colony stimulating factor 1 (CSF1; also known as M-CSF) for their development and/or survival, CD64^−^ CD11c^+^ MHCII^+^ MPs, but not CD64^+^ MPs, are highly dependent on the cDC-specific growth factor Flt3L (10, 11). Consistently, CD64^−^ CD11c^+^MHCII^+^ MPs have been shown to continually migrate to the mesenteric lymph nodes in a CCR7-dependent manner to participate in T cell priming (10, 12–14), defining features of cDC. In contrast, CD64^+^ MPs are non-migratory and display characteristic macrophage morphology, with abundant cytoplasm and cytoplasmic vacuoles (9, 15, 16). Thus, by multiple criteria, CD64^−^ CD11c^+^ MHCII^+^ MPs and CD64^+^ MPs fit the definition of cDC and macrophages, respectively. One additional feature of murine intestinal macrophages that distinguishes them from cDC is their high expression of the chemokine receptor CX3CR1 (9, 15, 17–20). Indeed, by using *Cx3cr1*^+/gfp^ knock-in mice (21), mature CX3CR1^hi^ macrophages can be visualized throughout the lamina propria (LP), the large layer of connective tissue underlying the epithelium, as well as in the deeper layers of the gut wall, such as the submucosa and muscularis (17, 19, 20). Macrophages in these distinct sites are reported to express differential levels of CD11c, with CD11c^+^ and CD11c^−/lo^ CX3CR1^hi^ macrophages enriched in the LP and muscularis, respectively (18, 20, 22). As discussed in more detail below, additional heterogeneity has been unmasked recently by transcriptional profiling, with discrete subsets of CX3CR1^hi^ macrophages identifiable based on their expression of CD4 and Tim4 (23, 24).

The recent advances in multi-parameter analysis have also led to the much-needed alignment of analysis of murine and human tissue macrophages. The use of markers such as CD64 and CD14 has meant that the same cells can be characterized across species (7, 9, 10, 25). This has highlighted similarities, but also important differences between mouse and man. For instance, expression of CD4, CD163, CD172a (SIRPα), and CD206 are conserved features of intestinal macrophages across species (7, 9, 23, 24, 26). However, mature intestinal macrophages in humans express only low levels of the CX3CR1 and CD11c markers found on their murine equivalents. Very recent work has also described potential phenotypic heterogeneity between human LP and muscularis macrophages, with the latter expressing higher levels of CD14 and CD11b (7).

## Functions of macrophages in intestinal homeostasis

**Figure 1:**
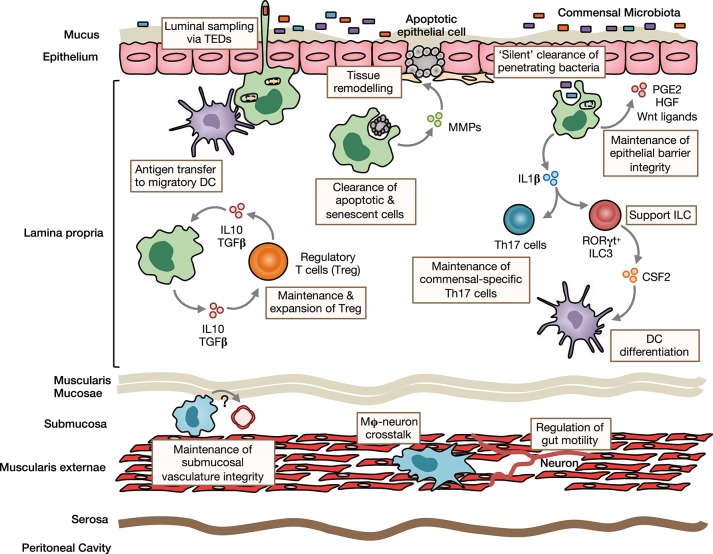
Homeostatic functions of intestinal macrophages. Intestinal lamina propria (LP) macrophages are highly phagocytic and are responsible for clearing apoptotic and senescent epithelial cells. Through their expression of tissue-remodeling metalloproteinases and secretion of factors that stimulate epithelial stem cell renewal, such as prostaglandin E2 (PGE2), hepatocyte growth factor (HGF) and Wnt ligands, they promote epithelial integrity. Their position under the epithelial monolayer and their bactericidal activity, mean LP macrophages are ideally placed to capture and destroy any bacteria that breach the barrier. They may also send cellular processes across the epithelial barrier to sample luminal contents. Macrophages can transfer acquired antigen to migratory dendritic cells (DCs) for presentation to T cells in the draining mesenteric lymph nodes. Through their production of immunoregulatory cytokines, such as IL10 and TGFβ, they maintain and facilitate secondary expansion of regulatory T cells (Tregs) locally in the LP. In a similar manner, they support Th17 cells and ILC3s through their production of IL1β, which is induced by exposure to the microbiota or its derivatives. Macrophages are also present in deeper layers of the gut wall, including the submucosa and muscularis externae. Submucosa macrophages are thought to support the integrity of the submucosal vasculature, although the factors involved in this interaction remain unclear. Muscularis macrophages participate in bidirectional crosstalk with sympathetic neurons of the enteric nervous system and influence gut motility.

Macrophages play a variety of roles to maintain intestinal homeostasis (Figure 1). Like their counterparts in other tissues, macrophages in the gut wall are avidly phagocytic. However, while being highly bactericidal, phagocytosis by intestinal macrophages does not result in an overt inflammatory response in both mouse and man (see below)(7, 9, 25, 27, 28). Consistent with this role, intestinal macrophages display high expression of genes associated with phagocytosis, such as *Mertk, Cd206, Gas6, Axl, Cd36, Itgav*, and *Itgb5* (23, 29). Integrins αv and β5 dimerise to form αvβ5, which is involved in the uptake of apoptotic cells (29), a process known as efferocytosis (30). Notably, *Lys2*-directed deletion of integrin αv results in the accumulation of apoptotic cells in the intestine (31), and *Itgb5* deficiency predisposes to increased susceptibility to DSS-induced colitis (29), highlighting a particularly important role for this pathway in this process.

The sub-epithelial positioning of LP macrophages means they are ideally placed to capture and eliminate any bacteria that cross the epithelial barrier. In addition, murine studies have shown that they are able to sample luminal bacteria, involving the formation of transepithelial dendrites (TEDs), cellular processes that cross the epithelial barrier without perturbing tight junctions and epithelial integrity and depend on the CX3CL1-CX3CR1 axis (32–34). A similar process may allow mature CX3CR1^hi^ macrophages in the upper small bowel to capture dietary materials and is suggested to be involved in the generation of oral tolerance to dietary antigens (35). This requires the induction of antigen specific Tregs in the gut draining mesenteric lymph nodes with gut homing properties (36, 37). Given that CX3CR1^hi^ macrophages do not migrate to draining lymph nodes under normal conditions and naïve T cells are essentially absent in the LP (13, 15, 38), they are unlikely to play a major role in this process. However, they may contribute to the induction of oral tolerance through antigen transfer to migratory CD103^+^ DC via connexin-43-dependent gap junctions (35). Indeed, mice that lack connexin-43 in CD11c^+^ cells fail to develop oral tolerance (35). Macrophages have also been proposed to regulate oral tolerance development by supporting Treg maintenance locally in the mucosa 39, 37, 40. This is thought to involve macrophage-derived IL10, as *Cx3cr1*-mediated deletion of IL10 reduces antigen specific Treg frequencies in a model of oral tolerance (37, 40). Interestingly however, deletion of IL10 in macrophages appears to have no impact on the overall abundance of endogenous Treg (40, 41). In addition to Tregs, macrophages may also support the induction/maintenance of commensal-specific Th17 cells through IL1β secretion (42, 43). Notably, whether maintenance of mucosal T cells also involves cognate interactions remains to be determined with certainty. Although macrophages are very poor in activating naive T cells compared to DCs (15), their high expression of MHCII suggests that they might be involved in antigen presentation to previously activated T cells locally in the intestine. By doing so, they could maintain or promote further differentiation of antigen-specific T cells. Consistent with this, *Cx3cr1*-mediated deletion of MHCII retards the generation/maintenance of antigen-specific Treg after feeding of ovalbumin (OVA) (40). Macrophages may also influence T cell priming indirectly through their effects on cDC differentiation. For instance, secretion of IL1β has been shown to enhance ILC3 production of CSF2 (44), which is known to control cDC differentiation in the intestinal LP (45). Notably, most functional analyses have been performed in mice and whether human intestinal macrophages carry out the same roles remains unclear.

While it has been known for many years that macrophages are present in deeper layers of the gut wall (46), only recently has work begun to interrogate their role in intestinal homeostasis. Macrophages in the muscularis are intimately associated with the enteric nervous system and, in mice, appear morphologically and transcriptionally distinct (47). There is bidirectional crosstalk between muscularis macrophages and neurons, where macrophage-derived bone morphogenic protein 2 (BMP2) acts on the BMP receptor (BMPR) expressed by enteric neurons to induce secretion of CSF1, which maintains the muscularis macrophage compartment and stimulates further BMP2 expression (20, 22). These interactions regulate smooth muscle contractions, thereby controlling peristalsis, and can be disrupted by broad spectrum antibiotics (22), suggesting the microbiota may regulate gut motility to some extent (48). Macrophages are also found in the submucosa and recent depletion studies have revealed a role for these cells in maintaining the integrity of the submucosal vasculature (47). Thus, macrophages fulfill niche-specific functions to meet the local demands of their microenvironment.

## Origin of intestinal macrophages

There have been major developments in our understanding of macrophage ontogeny in recent years [see (49) for review]. Traditionally, it was proposed that tissue macrophages were derived from blood monocytes that were replenished in turn by highly proliferative bone marrow (BM) progenitors as part of a linear mononuclear phagocyte system (MPS) (50, 51). Geissmann et al. (52) then refined this model demonstrating that the murine monocyte compartment, like its human counterpart (53), is heterogeneous, with subsets defined on the basis of Ly6C expression. In this scheme, Ly6C^hi^ monocytes were shown to preferentially enter tissues under inflammatory conditions, leading to them being described as “inflammatory” monocytes (52). However, as discussed below, Ly6C^hi^ monocytes can also be found in healthy tissues and tend to fulfill the functions classically ascribed to monocytes, therefore these are now referred to as “classical” monocytes. Because they did not enter inflamed tissues, it was proposed that Ly6C^lo^ monocytes were the precursors of tissue resident macrophages (52). However, adoptively transferred Ly6C^lo^ monocytes rarely enter healthy tissues, even following diphtheria toxin (DT)-mediated depletion of resident macrophages (9, 17). Moreover, recent work has shown that a major function of ‘non-classical' Ly6C^lo^ monocytes is to patrol the vasculature and scavenge necrotic endothelial cells (54) rather than acting as a circulating intermediate. Thus, in some respects, Ly6C^lo^ monocytes could be thought of as macrophages of the circulatory system.

Rather than deriving from blood monocytes, recent elegant fate mapping techniques have shown that many tissue macrophages exist independently from conventional haematopoiesis and instead derive from embryonic precursors arising from the yolk sac and/or fetal liver (55–59). For instance, microglia of the central nervous system and epidermal Langerhans cells appear to maintain themselves autonomously through intrinsic longevity and *in situ* self-renewal throughout adult life (60–64). In contrast, we have shown that although the intestine is initially seeded by embryo-derived macrophages, these are subsequently displaced with age by cells deriving from conventional haematopoiesis (16). Consistently, colonic macrophages, but not microglia or Langerhans cells, are labeled in genetic fate mapping studies exploiting *Flt3* or *Kit* expression to fate map cells deriving from haematopoietic stem cells (HSCs) (16, 55, 59, 65). Moreover, intestinal macrophages are largely replaced by donor cells in the setting of parabiosis and in tissue-protected bone marrow chimeric mice, unlike many other tissue macrophages (16, 56, 66–69). The finding that macrophage numbers are reduced in the gut wall of unmanipulated adult *Ccr2*^−/−^ mice (9), in whom classical Ly6C^hi^ monocyte egress from BM is defective (70), implies that Ly6C^hi^ and not Ly6C^lo^ monocytes are the main precursors of intestinal macrophages in adulthood. In line with this, adoptively transferred classical Ly6C^hi^ monocytes, but not Ly6C^lo^ monocytes, give rise to fully mature intestinal macrophages (9, 17, 19, 71). Furthermore, intestinal macrophages are eliminated by repetitive administration of DT to CCR2-DTR transgenic mice (43, 72), again indicating that the macrophage pool relies on CCR2-dependent replenishment. Notably, as well as its role in BM egress, homeostatic extravasation of Ly6C^hi^ monocytes from the bloodstream into the intestinal mucosa relies on the CCL2-CCR2 axis. This is demonstrated by the failure of both WT monocytes to enter the colonic mucosa of *Ccl2*-deficient mice and *Ccr2*-deficient monocytes to enter the mucosa of WT mice in mixed BM chimeras or in the setting of parabiosis (5, 16, 73).

**Figure 2:**
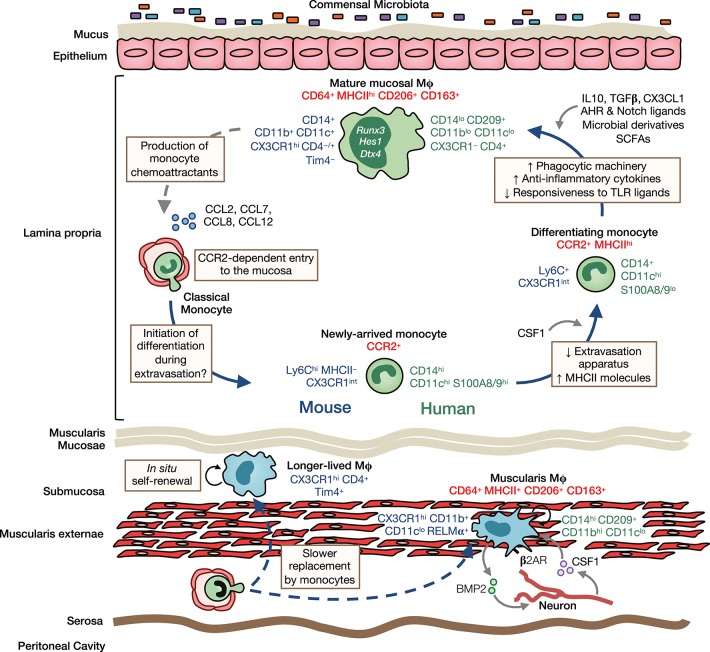
Heterogeneity, origin and differentiation of intestinal macrophages. The majority of mucosal macrophages are replenished by classical monocytes that enter the mucosa in a CCR2-dependent manner and differentiate through a series of intermediaries (mouse-and human-specific markers denoted in blue and green, respectively) to give rise to mature macrophages, which can be identified in both mouse and man as CD64^+^MHCII^hi^ CD206^+^CD163^+^ cells (common markers denoted in red). In addition, high levels of CD11b, CD11c, CD14, and CX3CR1 are characteristic features of murine LP macrophages. In contrast, human LP macrophages express only low levels of most of these markers but express high levels of CD209. Once in the mucosa and under cues from the local environment, monocytes first upregulate MHCII and downregulate molecules involved in extravasation, such as CCR2, LFA-1 and CD62L. They then upregulate phagocytic receptors and increase their production of anti-inflammatory cytokines, as well as becoming hyporesponsive to stimulation through e.g., TLRs. Studies in mice have identified IL10, TGFβ, and CX3CL1 as key factors in promoting macrophage differentiation in the healthy mucosa. Furthermore, exposure to the microbiota and its metabolites is known to influence macrophage differentiation and the rate of their turnover in the LP. Mature macrophages may also regulate their own turnover through secretion of monocyte chemoattractants, such as CCL2, CCL7, CCL8, and CCL12. Longer-lived macrophages may also exist in the murine intestinal mucosa and submucosa, and can be identified by their expression of the phagocytic receptor Tim4. While sharing certain features with their LP counterparts, such as CD64, MHCII, CD206, and CD163 expression, muscularis macrophages have a relatively distinct phenotype. In mouse, they express low levels of CD11c but high levels of the immunoregulatory cytokine RELMα, whereas in man, they have high levels of CD14 and CD11b. Muscularis macrophages are acutely dependent on CSF1 and norepinephrine signaling by sympathetic neurons via β2 adrenergic receptors (β2AR) shapes their differentiation. Monocytes also replenish macrophages of the muscularis, although the rate of replenishment is slower than in the mucosa and a larger proportion of these macrophages are long-lived.

It is now clear that a monocyte to macrophage differentiation continuum exists in the intestinal LP, a process that has become known as the monocyte “waterfall” (5, 9) (Figure 2). At one end are Ly6C^hi^ CX3CR1^int^ MHCII^−^ (“P1”) monocytes that appear phenotypically and morphologically similar to their counterparts in blood. Indeed, monocytes in the mucosa retain expression of molecules involved in chemotaxis and extravasation from the circulation, such as CCR2, CD62L, VLA-1, LFA-1, and of course Ly6C (23). These monocytes first acquire MHCII expression (‘P2' monocytes), before downregulating Ly6C, and the other markers of extravasation (“P3”macrophages), and finally upregulating CX3CR1 to give rise to fully mature (“P4”) macrophages; this process takes around 5–6 days and involves major gene expression changes (5, 9, 23). Importantly, there is mounting evidence that an analogous “waterfall” is present in the human intestinal mucosa, with classical CD14^hi^CCR2^+^CD11c^hi^ monocytes at one end and mature CD14^lo^CCR2^−^CD11c^lo^ macrophages at the other (7, 9, 25) (Figure 2). In support of this, Bujko et al. have recently used HLA-mismatched duodenal transplants to measure turnover of intestinal macrophages in man, showing that donor CD14^hi^CCR2^+^CD11c^hi^ cells in the graft, which are analogous to P1/P2 cells in mouse, are rapidly replaced by recipient cells. Mature macrophages are also replaced by recipient-derived cells, albeit at slower rates (7). This contrasts markedly with Langerhans cells of the skin epidermis, which have been shown to remain of graft origin in transplanted skin for at least up to 10 years (74). Similarly, alveolar macrophages persist for up to 2 years in transplanted lungs (75). Thus, the limited data available from human transplant studies support the findings from fate mapping studies in mice (64, 65, 69, 76).

Despite the evidence that intestinal macrophages are derived from continuous replenishment by extravasating monocytes, this idea may need to be refined on the basis of very recent findings that long-lived macrophages may be present in the adult intestine (24, 47). Longitudinal fate-mapping using *Cx3cr1*-based strategies and tissue-protected BM chimeric mice have identified macrophages that persist for longer than 8 months in the gut wall (47). Two independent studies showed that long-lived macrophages can be identified by Tim4 and CD4 expression (24, 47), which are unaffected by *Ccr2* deficiency, unlike most of their Tim4^−^ counterparts (24). This is consistent with the long-lived nature of Tim4-expressing macrophages in other tissues, such as the liver and the peritoneal cavity (69, 77). Notably, De Schepper and colleagues (47) showed that long-lived macrophages were predominantly found in the deeper layers of the gut wall, such as the muscularis and submucosa, whereas mucosal macrophages showed high levels of turnover from BM. Importantly, this group also showed that long-lived macrophages derived from both embryonic and BM-derived cells, demonstrating that intrinsic longevity is not an exclusive property of embryo-derived macrophages (47). Thus, in light of these findings, it is clear that the origin of intestinal macrophages is highly dynamic, with embryonic and BM-derived macrophages present alongside one another in each layer of the gut wall, the proportions of which change markedly with age and microbial colonization in a niche-specific manner (see below). Indeed, this brings the gut into line with other tissues, such as the heart (58, 78, 79), lung (65, 69, 80), dermis (67), and the peritoneal cavity (69, 81, 82) where short-lived and long-lived macrophages co-exist.

Here it should also be noted that it has never been shown definitely that patrolling Ly6C^lo^ monocytes cannot contribute to gut macrophage replenishment. One approach has been to assess macrophage abundance in the gut wall of mice in whom Ly6C^lo^ blood monocytes are markedly reduced, for instance those deficient in *Cx3cr1* (37, 83). However, different groups have reached discordant conclusions on the effect of *Cx3cr1* deficiency on intestinal macrophage numbers (37, 83). Furthermore, given the high expression of CX3CR1 by intestinal macrophages themselves, effects of *Cx3cr1* deficiency may be due to altered differentiation and/or survival of mature macrophages rather than indicating derivation from Ly6C^lo^ monocytes (see below). More recent work has identified *Nr4a1* as a master regulator of Ly6C^lo^ monocyte differentiation and survival (84). New tools that specifically target *Nr4a1* deficiency to monocytes, while sparing its roles in macrophage function, such as those described recently by Hedrick and colleagues (85), will be critical to assess the role of Ly6C^lo^ monocytes in tissue macrophage replenishment under normal physiological conditions and if this changes in the context of disease.

### What controls the origin of intestinal macrophages?

The exact factors that determine why different tissues contain macrophages of distinct origins remain very poorly understood. Specifically, it is unknown how and why embryonic-derived macrophages persist in the CNS and the epidermis, but fail to persist in significant numbers in the gut mucosa. It has been proposed that this could simply reflect niche accessibility and availability (86) and indeed, there is free accessibility to the mucosa throughout life, whereas the brain and epidermis are separated from the vasculature during development by the blood brain barrier and basement membrane, respectively. However, other tissue macrophages that are not separated from the vasculature by a physical barrier, such as liver Kupffer cells, also exist relatively autonomously, suggesting that tissue accessibility may not be the main factor influencing replacement by blood monocytes (65). In the intestine, monocyte recruitment may be driven by the “physiological inflammation” generated by exposure to antigenic material from the diet or commensal bacteria (87). Indeed, there are now several lines of evidence to demonstrate a key role for the microbiota in influencing macrophage population dynamics in the mucosa. First, major changes in the colonic macrophage compartment are seen following microbial colonization, particularly at the point of weaning where monocyte differentiation through the monocyte “waterfall” becomes established (16). Secondly, macrophage turnover can be reduced by administration of broad spectrum antibiotics, further indicating a role of the commensal microbiota in controlling macrophage turnover (16). Moreover, fewer macrophages are found in the gut wall of germ free mice compared with their SPF counterparts(16, 24, 88). The mucosal microenvironment may actually programme macrophages to orchestrate their own replacement. This is supported by the findings that as intestinal macrophages mature, they progressively upregulate monocyte chemoattractants, such as CCL7, CCL8, and CCL12 (23). As noted above, the microbiota may constitute one stimulus for this differentiation and additional possibilities could include dietary metabolites or the continual mechanical stress generated by peristalsis. Mechanical stress has been suggested to explain the replacement of embryo-derived macrophages in the heart (89) and as well as generating low grade “inflammation,” it could simply prevent long term macrophage residence. That differential turnover rates of macrophages are observed in distinct anatomical locales of the gut wall could reflect the fact that particular niches do not support macrophage self-renewal. However, whether distinct macrophage subpopulations display differential rates of proliferation has not been tested experimentally. Thus, while some progress has been made in understanding macrophage turnover dynamics, more work is needed to identify the factors that govern this process.

## Environmental programming of intestinal macrophages

While it is clear that monocytes progress through a defined series of intermediaries to replenish the majority of macrophages in the gut, the molecular factors in the gut environment that imprint the unique phenotypic and functional profile of intestinal macrophages are only starting to be understood. CSF1 is clearly involved in the differentiation and/or survival of intestinal macrophages, as demonstrated by their reduction in *Csf1*^op/op^ mice, which have a naturally occurring inactivating mutation in the CSF1 gene (90, 91), their inability to arise from *Csf1r*^−/−^ precursors in a competitive BM chimeric setting (11) and their depletion by anti-CSF1R antibody treatment (22, 92, 93). As described above, upregulation of MHCII is one of the first features of monocyte differentiation in the mucosa of mouse and man (5, 7, 9). Although it is clear that this occurs independently of the IFNγ-STAT1 pathway (94), the exact factors that drive upregulation of MHCII remain unclear. Given that this appears to be a common feature of monocytes entering a variety of tissues (16, 59, 67, 68, 95), it is plausible that MHCII expression may be triggered by extravasation through the vascular endothelium (68). Indeed monocytes in the colonic mucosa already display major transcriptional differences compared with their counterparts in blood (23), despite appearing phenotypically similar. This is tissue specific, because recently arrived colonic monocytes are also transcriptionally distinct from their phenotypic counterparts in the dermis (23). Once in the mucosa, we have shown that TGFβR signaling is essential for the terminal differentiation of macrophages. In particular, upregulation of genes associated with the homeostatic profile of gut macrophages, such as CX3CR1, IL10, and αvβ5 integrin relies on the TGFβ-TGFβR axis (23). Consistent with this, expression of the Runt-related transcription factor 3 (RUNX3), which regulates TGFβ signaling, is a unique feature of intestinal macrophages (96). The TGFβ-TGFβR axis may also regulate macrophage turnover by dampening expression of the monocyte chemoattractant CCL8 by colonic macrophages (23). Although many sources of TGFβ exist in the mucosa, macrophages themselves may be important, since efferocytosis is known to induce TGFβ expression in macrophages (97) and, at least in man, macrophages may activate TGFβ through their expression of integrin β8 (98). Indeed, uptake of apoptotic epithelial cells induces an anti-inflammatory programme in intestinal macrophages (99). The epithelium may also support macrophage differentiation through expression of Notch ligands, such as Delta-like and Jagged family members, as mature macrophages express high levels of *Hes1* (23), a downstream target of Notch signaling, and their differentiation is disrupted when Notch signaling is ablated (100).

A characteristic feature of mature intestinal macrophages is their hyporesponsiveness to exogenous stimulation (9, 27, 101–105), a functional adaptation that allows these cells to exist in this microbe-rich environment. Interestingly, TGFβ does not appear to be responsible for the unresponsiveness of intestinal macrophages to TLR stimulation in mice (23), whereas this is proposed to be a key role of TGFβ in the human mucosa (102, 105). In contrast, the IL10-IL10R axis plays a fundamental role in the control of macrophage responsiveness in both species. Colonic macrophages from mice in which this axis has been disrupted, either globally or specifically in myeloid cells, have heightened expression of proinflammatory mediators, such as iNOS, IL23, and IL12. As a result, they display overt responsiveness to TLR stimulation and an altered metabolic profile, leading to the development of spontaneous intestinal inflammation (41, 103, 106–109) that can be rescued by rendering macrophages unresponsive to TLR stimulation through cell specific deletion of the TLR adaptor molecule MyD88 (110). Early onset IBD occurs in patients with polymorphisms in *IL10RA* and *IL10RB* genes (111), and *in vitro* generated monocyte-derived macrophages from these patients respond aggressively to LPS stimulation (108). The exaggerated pro-inflammatory responses in the absence of IL10R signaling may result from a failure to downregulate inflammation potentiating molecules such as TREM-1 and STAT1 (41, 112) and/or altered accessibility to pro-inflammatory genes that is normally restricted by IL10-dependent chromatin remodeling (113, 114). IL10 can also limit pro-inflammatory responses by inducing expression of negative regulators of NF-kB, such as IBNS (115).

The high expression of CX3CR1 by murine intestinal macrophages and their positioning adjacent to CX3CL1-producing epithelial cells suggests that the CX3CL1-CX3CR1 axis could also control macrophage differentiation. For instance, CX3CR1 is indispensable for the formation of TEDs that permit luminal sampling by LP macrophages (32–34). Furthermore, *Cx3cr1*-deficient macrophages produce less IL10 (37), suggesting the CX3CL1-CX3CR1 axis promotes the regulatory features of gut macrophages. In line with this, *Cx3cr1*-deficient mice have been shown to be more susceptible to chemically-induced colitis (83), although this has been contested by other reports showing that *Cx3cr1* deficiency suppresses DSS-induced and T cell transfer colitis (32, 34). However, as noted above, human intestinal macrophages do not express CX3CR1, raising questions about the general significance of its role.

In addition to influencing their turnover (16, 24), the microbiota is required for optimal production of IL1β (42) and IL10 by intestinal macrophages(40, 71, 103), with the latter proposed to rely on autocrine type 1 IFNs (116). Microbial colonization may also contribute to the anergic phenotype of colonic macrophages, since some studies have shown them to display TLR hyperresponsiveness when isolated from germ free mice (103), although this is disputed by others (104). The microbiota may act directly on macrophages, for example through the release of as yet unidentified polysaccharides, such as that recently identified by the Powrie group to be released by *H. hepaticus* (117) or via metabolism of dietary fiber to provide short chain fatty acids (SCFAs), which are known to have wide ranging effects on immune cell function (118). In particular, the SCFA butyrate can repress *Il6, Il12b*, and *Nos2* expression by colonic macrophages (119) and alter their metabolic profile (120), while propionate can dampen macrophage activation *in vitro* (121). Aryl hydrocarbon receptor (Ahr) ligands derived from the microbiome or the diet may also control macrophage behavior. Consistent with this idea, CD11c^Cre^-*Ahr*^fl/fl^ mice display heightened susceptibility to DSS-induced colitis, which is attributed to altered Wnt ligand expression by AhR-deficient macrophages and impaired epithelial barrier integrity (122). Thus, multiple environmental factors act in concert to control macrophage differentiation and function in the mucosa.

The equivalent factors controlling macrophage differentiation in the muscularis remain relatively unexplored, although it is clear they are acutely dependent on CSF1R signaling (22). In addition, norepinephrine signaling by sympathetic neurons via β2 adrenergic receptors on muscularis macrophages has been reported to shape their tissue protective phenotype (20). Notably, the abundance and patterning of macrophages in the muscularis is not dependent on neuronal signals because they are normal in *Ret*^−/−^ mice, which lack an enteric nervous system, as well as in patients with Hirschsprung disease (HSCR), where the enteric nervous system is absent from the distal bowel (123).

## Monocytes and macrophages in intestinal inflammation

The monocyte/macrophage compartment alters markedly in both CD and UC, with accumulation of CD14^hi^CD11c^hi^ monocytes/immature macrophages that come to outnumber CD64^+^HLA-DR^hi^CD14^lo^ resident macrophages (9, 27, 124,–127). In contrast to their homeostatic counterparts, these CD14^hi^ cells in the gut produce pro-inflammatory cytokines and chemokines, such as TNFα, IL1β, IL6, IL12, IL23, and CCL11 (125, 127), display respiratory burst activity (128) and respond in an aberrant manner to commensal bacteria (125). In addition, they express high levels of TREM1, which can potently amplify pro-inflammatory responses (129). Importantly, mucosal healing in IBD patients receiving anti-TNF has been shown to be accompanied by loss of these CD14^hi^ cells and accumulation of CD206^+^ macrophages, which are thought to be pro-reparative (130). Although anti-TNF (adalimumab) has been shown to bind membrane-bound TNF on CD14^+^ intestinal macrophages in CD patients (131, 132), whether this triggers a phenotypic switch of existing pro-inflammatory macrophages or if these are replaced by CD206^+^ macrophages remains unclear. Thus, much of the recent work in this field has focussed on understanding the relationship between homeostatic and pro-inflammatory macrophages and the nature of their precursors, with the ultimate aim of identifying novel therapeutic targets.

Several models of intestinal inflammation, including T cell transfer, *Helicobacter hepaticus*-induced and DSS-induced colitis have been used to dissect these processes experimentally. As in humans, all these models show intense accumulation of classical (Ly6C^hi^) monocytes, together with their immediate progeny that express intermediate levels of CX3CR1 (P1, P2, P3 subsets—see above) (5, 9, 19, 71, 93, 133–135). These cells respond in a highly pro-inflammatory manner to TLR stimulation, express reactive oxygen intermediates, produce high levels of IL1β, IL6, IL12, IL23, and TNFα and express high levels of TREM1 (5, 9, 17, 19, 71, 135), again mirroring the processes seen in human IBD. In contrast to these effects on monocytes, the CX3CR1^hi^ resident macrophages that persist in colitis retain their anti-inflammatory signature (9, 19, 134), suggesting they may continue to play an immunoregulatory role even during inflammation (136).

Multiple lines of evidence indicate that Ly6C^hi^ monocytes and their derivatives are of crucial importance in the intestinal pathology. Firstly, neutralization of IL1β, which is thought to arise predominantly from elicited monocytes, reduces susceptibility to chemically-induced colitis (137). Secondly, colitis development is reduced by selective ablation of *Tnfa* in Ly6C^hi^ monocytes (17). Whether this reflects direct effects of monocyte-derived TNFα in tissue pathology is uncertain, as monocyte survival appears to require autocrine TNFα (138), suggesting that reduced monocyte accumulation in the gut may be the mechanism underlying protection by depletion of TNFα. Finally, mice in whom monocyte recruitment to the inflamed mucosa is defective due to deletion or neutralization of CCL2, CCR2 or β_7_ integrin are protected from DSS-induced colitis (9, 19, 73, 133, 139, 140). Importantly, the CCL2-CCR2 axis may also govern monocyte migration in man, where classical monocytes also express CCR2 (141) and elevated levels of its ligands CCL2 and CCL4 are found in IBD mucosa (142). Furthermore, radio-labeled CD14^hi^ classical monocytes have been shown to migrate to actively inflamed regions of IBD mucosa (124). As in the healthy gut, resident macrophages may contribute to this recruitment of monocytes through the release of CCR2 ligands. Nevertheless, it is important to note that CCR2 may not govern monocyte migration in all contexts, as accumulation of Ly6C^hi^ monocytes and their progeny is unaffected by CCR2 deficiency in *H. hepaticus* induced colitis (110) and CCR1 plays a key role in monocyte migration during acute toxoplasmosis (143). Moreover, circulating monocytes in mouse and man express CCR5 (144), which is known to navigate monocytes in certain contexts of inflammation (145), and CCR5 deficient mice develop less inflammation when administered DSS (146). In addition, a unique CD169^+^ subset of CX3CR1^hi^ macrophages, located preferentially around intestinal crypts, is expanded during experimental colitis and is important for pathogenesis via its ability to recruit monocytes through secretion of the CCR2/CCR3/CCR5 ligand CCL8 (147, 148).

As well as direct effects of elicited monocytes and their products, these cells can recruit and support other innate and adaptive immune effector cells that are important in pathology. For instance, CD14^hi^ monocytes/macrophages in the IBD mucosa are thought to support pathogenic T cell function through IL23 production and their expression of CD40 and CD80 (125, 149, 150). Consistent with this idea, CX3CR1^int^ monocyte/macrophage-derived IL23 supports effector T cell differentiation during *H. hepaticus*-induced colitis (93, 110, 134), assisting the generation of highly pathogenic Th17 cells that co-express IFNγ (93, 151). Elicited monocytes/macrophages may also recruit eosinophils to the inflamed mucosa through the production of CCL11, although whether these play a pro-inflammatory or pro-resolution function remains unclear (127, 152–154).

### Macrophages in intestinal infection

Despite their pathogenic role in sterile intestinal inflammation, Ly6C^hi^ monocytes and their progeny are vital for protective immunity against enteric pathogens. For instance, *Ccr2*^−/−^ mice are more susceptible to infection with *Citrobacter rodentium*, a model of enteropathogenic and enterohaemorrhagic *E. coli* infection in man, and the protozoan parasite *Toxoplasma gondii* (155, 156). This can be restored by transfer of wild type Ly6C^hi^ monocytes. Although depletion of CCR2^+^ cells in the CCR2-DTR mouse leads to enhanced susceptibility to *C. rodentium* (157), it should be noted that this approach deletes both elicited and resident macrophages in the intestine (43, 72, 157), meaning the specific roles of these individual subsets cannot be distinguished in this model. Nevertheless, macrophages play an important protective role in *C. rodentium* infection via the production of IL1β, IL23, and TNF-like ligand 1A (TL1A), triggering IL22 production by ILC3s, which in turn augments local production of the anti-microbial proteins RegIIIβ and RegIIIγ (157–159), known to be necessary for *C. rodentium* clearance (160). Moreover, through their production of IL12 and IL23, macrophages support the differentiation of IFNγ and IL17-producing effector T cells (161). Whether this occurs exclusively in the mucosa or if Ly6C^hi^ monocyte-derived cells leave the mucosa to contribute to T cell priming in the lymph nodes remains a matter of debate and may depend on the nature of the inflammatory insult (162, 163). In addition to their pro-inflammatory roles, elegant work from the Belkaid lab has shown that Ly6C^hi^ monocytes can also exert regulatory functions. During acute toxoplasmosis, elicited Ly6C^hi^ monocytes respond to the microbiota by producing PGE2 and IL10 that protect against immunopathology by inhibiting neutrophil function (164). As a result, *Ccr2*^−/−^ mice show enhanced susceptibility to this model of infection (156). Thus, it is clear that monocytes play a multifaceted role in the inflamed mucosa.

Until now, we have considered macrophage function in Th1 and/or Th17-dominated forms of inflammation, but they also participate in the Th2-mediated protective immune responses generated against intestinal helminth parasites. However, the exact role macrophages play may depend on the parasite in question. For instance, while arginase producing, alternatively activated macrophages are critical for the expulsion of the gastrointestinal nematode *Heligmosomoides polygyrus bakeri* (165–167), inhibition of arginase has no effect on expulsion of *Trichuris muris* (168). Instead, macrophages are considered to play a more central role in the tissue repair that occurs after *T. muris* has been expelled. The role of alternatively activated macrophages in expulsion of the nematode *Nippostrongylus brasiliensis* also remains contentious (169, 170). Interestingly, although macrophage accumulation in Th2 type settings in other tissues is now typically thought to involve *in situ* proliferation of resident cells under the control of IL4 (171), accumulation of ‘alternatively activated' macrophages in the gut of mice with *T. muris* is dependent on monocyte infiltration (172). Thus, regardless of the nature of the insult, monocyte recruitment appears to be the principal mechanism of bolstering the macrophage reservoir in the gut mucosa.

### Monocyte differentiation in the inflamed mucosa

Why monocytes accumulate during colitis and do not differentiate into anti-inflammatory macrophages as they do in healthy tissue remains unclear. Based on adoptive transfer studies in the DSS-induced model of colitis, we proposed that immature monocytes accumulate due to a breakdown in the normal differentiation process (9). The exact cause of this remains elusive, but may reflect both a loss of factors that normally promote monocyte differentiation, such as IL10 and TGFβ, together with increased levels of pro-inflammatory cytokines that block this process or reduce monocyte half-life. Indeed, high levels of IFNγ are found in the IBD mucosa and have been shown to promote the pro-inflammatory features of CD14^+^ monocyte/macrophages (125). Consistent with this, deletion of IFNγR1 or its downstream signaling molecule STAT1 in mice limits the differentiation of pro-inflammatory Ly6C^+^MHCII^+^ monocytes in the colon and provides relative protection from DSS-induced colitis (94). IFNγ may also act by upregulating negative regulators of the TGFβR pathway, such as Smad7 (173), thus disrupting the pathway by which monocytes normally differentiate into mature, anti-inflammatory macrophages (see above). Finally, the hypoxic nature of the inflamed mucosa may support the differentiation of pro-inflammatory monocytes/macrophages, as myeloid-specific deletion of the hypoxia inducible factor (HIF)-1α also protects mice from DSS-induced colitis (174).

As well as local programming by the intestinal microenvironment, there is increasing evidence that monocytes arriving in the inflamed mucosa may be inherently different to those during health. Monocytosis is a feature of human and experimental IBD (175, 176), and monocytes arriving in the inflamed mucosa already have higher expression of TNFα, iNOS, IL6 and STAT1 compared with their homeostatic counterparts (9, 94). This may involve “priming” of monocytes in the BM by IFNγ derived from NK cells responding to IL12 released from the inflamed intestine, as has been shown to occur in acute toxoplasmosis (177), or through as yet unidentified pathways. Thus, the inflamed mucosal environment may control monocyte fate both locally and through long-range conditioning of BM precursors.

## Monocytes/macrophages during resolution of inflammation

Experimental models of colitis have also allowed characterization of the monocyte/macrophage compartment during the resolution of pathology. Cessation of DSS administration is accompanied by major changes in the macrophage pool, with a massive reduction in CX3CR1^int^ monocytes/macrophages and restoration of the CX3CR1^hi^ macrophage subset, together with loss of granulocytes (19). A similar contraction of inflammatory cells is seen following the infectious phase of *H. hepaticus*-induced colitis, although interestingly, eosinophils persist at elevated levels in this model, suggesting they may play a pro-resolution role (134). Resident intestinal macrophages promote mucosal healing, as colitis is worsened by their depletion (136) or if they are rendered unresponsive to anti-inflammatory cytokines, such as TGFβ (178). However, whether macrophages elicited by an inflammatory agent also play a pro-restorative role following removal its clearance remains unclear. Interestingly, resolution of inflammation in post-operative ileus is delayed in *Ccr2*^−/−^ mice, suggesting that recruited Ly6C^hi^ monocytes and their derivatives are important for restoration of homeostasis in the muscularis (179).

The fate of the monocytes elicited during inflammation in the repairing mucosa is unclear, although it is assumed that they are cleared by apoptosis, as in other tissues (180). This would be consistent with the increased numbers of apoptotic CD68^+^ cells seen in the healing mucosa of CD and UC patients treated with infliximab (181). An alternative fate of elicited monocytes is that they subsequently convert into mature resident macrophages under the guidance of local cues. While this has been shown to occur during the resolution of inflammation in other tissues, such as the peritoneal cavity (57), it is not known whether it occurs in the repairing mucosa. Moreover, just like in the setting of infection described above, long-range conditioning of monocytes may also occur during inflammation resolution meaning the nature of the monocytes arriving at the repairing mucosa may be intrinsically-distinct. Consistent with this idea, Ikeda et al. (182) have recently shown that a specific subset of Ly6C^hi^ monocytes expressing the regulatory molecule Ym1 can be found during the resolution phase of DSS-induced colitis and their depletion hinders effective mucosal healing.

## Conclusions and future perspectives

Several major advances have been made over the last few years in our understanding of intestinal macrophage ontogeny and development, including the identification of some of environmental signals that regulate tissue-specific phenotypes and functions. Nevertheless, many aspects of intestinal macrophage biology remain poorly understood. For instance, our understanding of heterogeneity within the intestinal macrophage compartment remains incomplete. The application of single cell technologies, such as single cell RNA sequencing, will continue to provide further insights into macrophage heterogeneity in both mouse and man. This should also allow for further alignment of the ways in which murine and human MPs are characterized and lead to better translation between systems. With the discovery of macrophage subpopulations, it will be important to determine the environmental signals that shape the phenotype, function and longevity of these niche-specific macrophages. Why do some niches promote the longevity of macrophages (e.g., the muscularis externa) whereas others (e.g., the LP) mainly rely on the constant replenishment by BM-derived monocytes? Understanding the cellular interactions between macrophages and their neighboring cells (e.g., stromal cells), the environmental challenges (e.g., antigenic exposure) in particular niches, as well as niche accessibility will be pivotal in answering this question. Importantly, macrophage longevity is not an exclusive property of embryo-derived macrophages and it remains to be determined whether long-lived embryo-derived and BM-derived macrophages perform analogous functions or if they have discrete roles in intestinal homeostasis. Another area warranting further investigation is how differences along the intestinal tract, for instance antigenic exposure and commensal microbiota composition, might impact macrophage development and function. A major effort must be placed on understanding how the monocyte/macrophage compartment changes during acute and chronic inflammation, as well as during inflammation resolution. Do long-lived macrophages persist during and following an inflammatory insult? If so, do they perform specific roles? Given that accumulation of pro-inflammatory monocytes/macrophages is a characteristic feature of IBD, it is vital to understand the precise nature of the molecular factors controlling monocyte/macrophage differentiation under normal physiological conditions, how these change during disease and the relative contribution of local conditioning vs. long-range effects on haematopoiesis. Providing answers to these questions will be vital if macrophages are to be realized as therapeutic targets in IBD.

## Author contributions

Both authors made a substantial, direct and intellectual contribution to the work, and approved it for publication.

### Conflict of interest statement

The authors declare that the research was conducted in the absence of any commercial or financial relationships that could be construed as a potential conflict of interest.
